# Genetically Predicted Peripheral Immune Cells Mediate the Effect of Gut Microbiota on Influenza Susceptibility

**DOI:** 10.3390/ijms25147706

**Published:** 2024-07-14

**Authors:** Shiqi Wang, Guosen Ou, Jialin Wu, Yaokang Chen, Lu Xu, Huachong Xu

**Affiliations:** School of Traditional Chinese Medicine, Jinan University, Guangzhou 510632, China; sikeiwong@stu2018.jnu.edu.cn (S.W.); ouguosen@stu2022.jnu.edu.cn (G.O.); wujialin@stu2020.jnu.edu.cn (J.W.); yiuhong@stu2022.jnu.edu.cn (Y.C.)

**Keywords:** Mendelian Randomization (MR), influenza, gut microbiota, immune cell, gut–lung axis

## Abstract

The communication mechanism of the gut–lung axis has received increasing attention in recent years, particularly in acute respiratory infectious diseases such as influenza. The peripheral immune system serves as a crucial bridge between the gut and the lungs, two organs that are not in close proximity to each other. However, the specific communication mechanism involving gut microbiota, immune cells, and their anti-influenza effects in the lung remains to be further elucidated. In this study, the effects of 731 species of peripheral immune cells and 211 different gut microbiota on influenza outcomes were analyzed using a two-sample Mendelian randomization analysis. After identifying specific species of gut microbiota and peripheral immune cells associated with influenza outcomes, mediation analyses were conducted to determine the mediating effects of specific immune cells in the protective or injurious effects of influenza mediated by gut microbiota. 19 species of gut microbiota and 75 types of peripheral immune cells were identified as being associated with influenza susceptibility. After rigorous screening, 12 combinations were analyzed for mediated effects. Notably, the down-regulation of CD64 on CD14- CD16- cells mediated 21.10% and 18.55% of the protective effect of *Alcaligenaceae* and *Dorea* against influenza, respectively. In conclusion, focusing on influenza, this study genetically inferred different types of gut microbiota and peripheral immune cells to determine their protective or risk factors. Furthermore, mediation analysis was used to determine the proportion of mediating effects of peripheral immune cells in gut microbiota-mediated susceptibility to influenza. This helps elucidate the gut–lung axis mechanism by which gut microbiota affects influenza susceptibility from the perspective of regulation of peripheral immune cells.

## 1. Introduction

Influenza viruses, belonging to the Orthomyxoviridae family and characterized by nucleic acids encapsulated in protein capsids, are significant causes of respiratory infections [1]. Influenza imposes a substantial socio-economic burden, with the influenza A virus responsible for 250,000–300,000 deaths and 3–5 million severe infections globally each year [2]. Hemagglutinin (HA) and neuraminidase (NA) are the major viral glycoproteins detected by antibodies, defining the virus subtypes [3]. The clinical outcomes of influenza infections range from mild upper respiratory illness to severe lower respiratory symptoms. Typical symptoms include sudden fever, muscle aches, headache, and fatigue, which peak 2–3 days post-infection and subside within 1–2 weeks [4]. Host defenses against viral infection involve both innate and adaptive immune responses. However, excessive or imbalanced immune responses can damage lung tissue, decrease respiratory capacity, and even death [5]. Vaccines are currently an essential means of preventing the onset of influenza, assisting the body in protecting against the virus through the production of specific antibodies. However, this protection tends to be weaker in the elderly and young children [6,7], and because influenza viruses are highly susceptible to antigenic drift and antigenic shift [8], it is necessary to explore complementary therapies to consolidate protection against influenza viruses.

The gut, the largest immune organ in the body, plays a crucial role in immune regulation. Studies of the gut–lung axis have increasingly highlighted the impact of gut microbiota interactions on asthma and various respiratory infections [9]. Firstly, the modulation of gut flora, including the application of probiotics and prebiotics has been found to help aid better protective efficacy of influenza vaccines. In addition, we are particularly interested in how the symbiotic gut microbiota modulates resistance to influenza viruses. The immune system, acting as a key bridge between the gut and the lungs, is of special interest. Microbial antiviral activity against influenza infection relies on the induction of type I interferon β [10], a critical mediator in immune homeostasis. Type I interferon restricts viral activity, promotes apoptosis in infected cells, and adjusts immune cell subsets [11]. Alternatively, the gut microbiota components of the gut modulate intestinal innate immunity, which affects the immune microenvironment of the lungs by modulating chemokines and causing the migration of immune cells, such as neutrophils and macrophages [2]. Innate immune cells, including macrophages and neutrophils, are key components in sensing infection and inhibiting viral replication early in the course of infection [12]. Complex and crucial links exist between the gut–lung axis and the microbiota–immunity axis in defending against influenza virus infection.

To explore the role of gut microbiota in influenza, randomized controlled trials (RCTs) that adequately control for confounding effects provide the highest level of evidence, although they can be expensive, time-consuming, and challenging. Retrospective, cross-sectional, or prospective studies, while easier to conduct, often fail to control for confounding factors. Furthermore, due to the complexity of the gut microbiota, strict control of a single bacterium to study the role of specific flora in influenza is difficult. This is because conventional microecological modulation therapies, such as oral probiotics or prebiotics, tend to exert a modulatory effect on multiple gut microbiota. On the other hand, for infectious diseases such as COVID-19 and influenza, studying their susceptibility factors is difficult due to the impossibility of anticipating the time of infection in patients and intervening in advance. Mendelian randomization (MR) analysis, a genetic inference method based on genome-wide association study (GWAS) data, enables the analysis of potential risk or protective factors for diseases. MR analysis uses genetic mutation loci strongly associated with exposure factors and the principle of random segregation and recombination of alleles during fertilization [13]. This method controls for confounders and examines the effect of exposure on outcome indicators, and enables the extrapolation of disease risk factors, which is difficult in the real world. As gut microbiota is influenced by environmental, dietary, and other confounding factors, MR analysis is particularly effective in characterizing its role in disease pathogenesis. However, for reliable results, the instrumental variables (IVs) used in MR analysis must meet three conditions: (i) the IVs must be strongly correlated with the exposure factor; (ii) the IVs must influence the outcome only through the exposure factor; (iii) the IVs must not influence the outcome through other confounding factors.

Ultimately, this study aims to enhance our understanding of the interactions between gut microbiota, immune regulation, and the mechanisms of influenza virus infection. By elucidating potential therapeutic targets and gaining deeper insights into influenza pathogenesis through advanced genetic inference techniques and the comprehensive analysis of immune cell traits, we hope to contribute to the design of strategies to prevent respiratory viral infections by modulating gut microbiota and peripheral immune cells, thereby reducing morbidity and preventing severe conditions.

## 2. Results

### 2.1. Two-Sample MR Analysis between Gut Microbiota and Influenza Outcome

**Figure 1:**
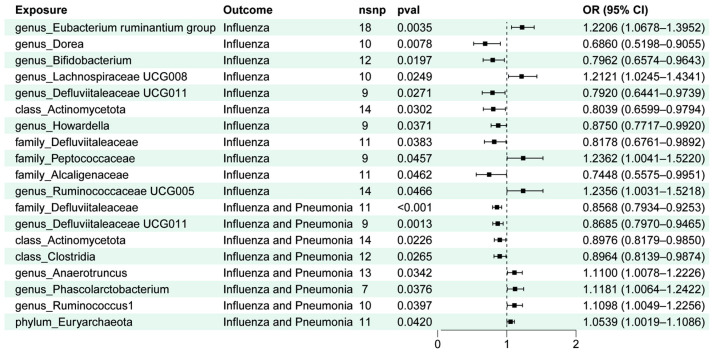
Result of two-sample MR analyses of gut microbiota taxa, “influenza”, and “influenza and pneumonia”.

A total of 19 gut microbiota (11 genera, 4 families, 3 classes, and 1 phylum) were identified as being associated with the occurrence of “influenza” or “influenza and pneumonia” (Figure 1). An increased abundance of some gut microbiota was linked to a lower risk of influenza and pneumonia, including *Defluviitaleaceae* (OR = 0.818, [95%CI: 0.676~0.989], *p* = 8.20 × 10^−5^), *Defluviitaleaceae UCG011* (OR = 0.792, [95%CI: 0.644~0.974], *p* = 1.32 × 10^−3^), *Actinomycetota* (OR = 0.804, [95%CI: 0.660~0.979], *p* = 2.26 × 10^−2^), and *Clostridia* (OR = 0.896, [95%CI: 0.814~0.987], *p* = 2.65 × 10^−2^). The abundance of certain gut microbiota was identified with a reduced risk of influenza, like *Dorea* (OR = 0.686, [95%CI: 0.520~0.905], *p* = 7.78 × 10^−3^), *Bifidobacterium* (OR = 0.796, [95%CI: 0.657~0.964], *p* = 1.97 × 10^−2^), *Defluviitaleaceae UCG011* (OR = 0.792, [95%CI: 0.644~0.974], *p* = 2.71 × 10^−2^), *Actinomycetota* (OR = 0.804, [95%CI: 0.660~0.979], *p* = 3.02 × 10^−2^), *Howardella* (OR = 0.875, [95%CI: 0.772~0.992], *p* = 3.71 × 10^−2^), *Defluviitaleaceae* (OR = 0.818, [95%CI: 0.676~0.989], *p* = 3.83 × 10^−2^), and *Alcaligenaceae* (OR = 0.745, [95%CI: 0.557~0.995], *p* = 4.62 × 10^−2^). Conversely, a number of gut microbiota are considered to correlate with a higher risk of developing influenza and pneumonia, containing *Anaerotruncus* (OR = 1.110, [95%CI: 1.008~1.223], *p* = 3.42 × 10^−2^), *Phascolarctobacterium* (OR = 1.118, [95%CI: 1.006~1.242], *p* = 3.76 × 10^−2^), *Ruminococcus1* (OR = 1.110, [95%CI: 1.005~1.226], *p* = 3.97 × 10^−2^), *Euryarchaeota* (OR = 1.054, [95%CI: 1.002~1.109], and *p* = 4.20 × 10^−2^). Moreover, the *Eubacterium ruminantium group* (OR = 1.221, [95%CI: 1.068~1.395], *p* = 3.47 × 10^−3^), *Lachnospiraceae UCG008* (OR = 1.212, [95%CI: 1.025~1.434], *p* = 2.49 × 10^−2^), *Peptococcaceae* (OR = 1.236, [95%CI: 1.004~1.522 ], *p* = 4.57 × 10^−2^), *Ruminococcaceae UCG005* (OR = 1.236, [95%CI: 1.003~1.522], and *p* = 4.66 × 10^−2^) are associated with a higher risk of influenza-onset (Figure 1).

### 2.2. Mediator Screening of Peripheral Immune Cells

**Figure 2:**
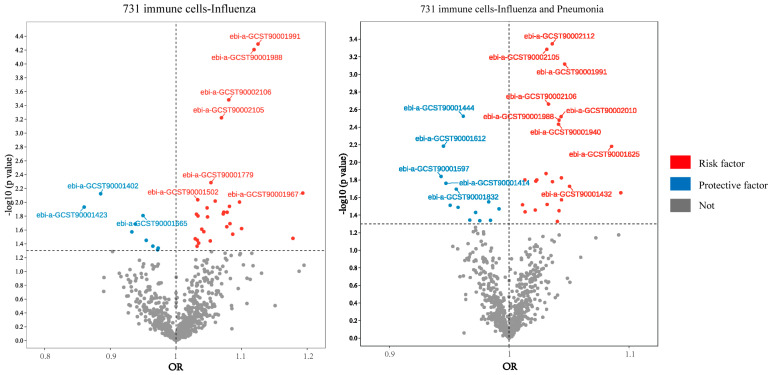
Immune cells associated with “influenza” and “influenza and pneumonia”; analysis based on two-sample MR analysis, IVW method.

**Figure 3:**
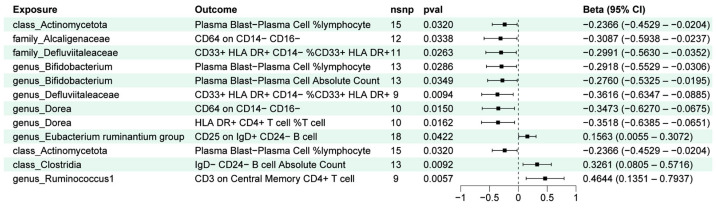
Positive results from the first step of mediation analysis: genetic inference between influenza-associated gut microbiota and peripheral immune cells.

**Figure 4:**
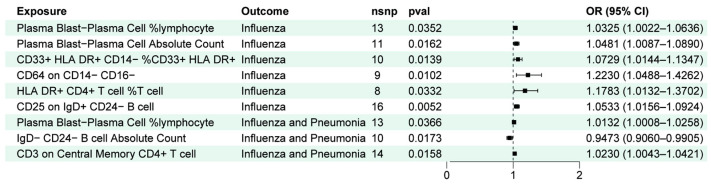
Positive results of the second step of the mediation analysis: genetic inferences between peripheral immune cells and influenza outcome.

To determine the immune cells relevant to mediating the effect of gut microbiota on influenza outcomes, we performed a two-step process. First, the impact of peripheral immune cells on “influenza” or “influenza and pneumonia” was assessed by mainly IVW methods, and a total of 38 species of peripheral immune cells were found to be associated with a risk of influenza, as well as 37 being related to the risk of influenza and pneumonia (Figure 2) (Supplementary Table). Second, we took the gut microbiota that were significantly correlated with influenza outcomes from the filter in 3.1 to make causal inferences with differential immune cells (Supplementary Table). After effect direction-screening, we enrolled 12 groups for mediated effects analysis (Figure 3 and Figure 4).

### 2.3. Analysis of the Mediating Effect of Peripheral Immune Cells in the Association between Gut Microbiota and Influenza Outcome

**Figure 5:**
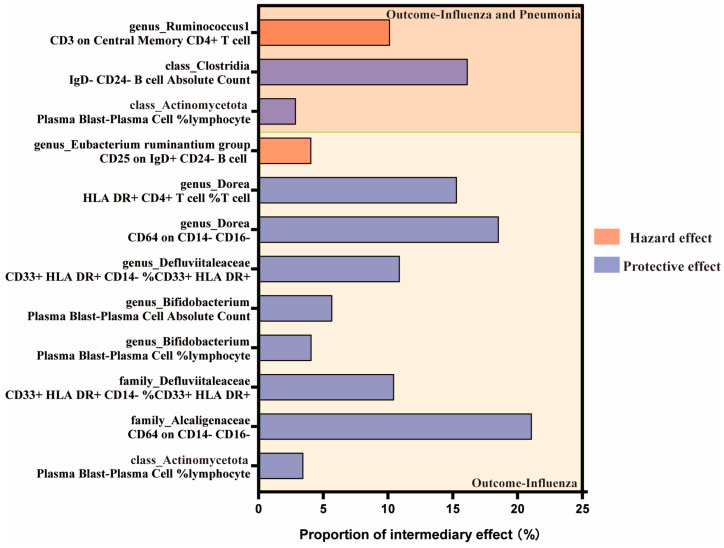
Proportion of mediating effects of peripheral immune cells between gut microbiota and influenza outcomes.

The results of the mediation analyses suggest that peripheral immune cells contributed to the effects between gut microbiota and the outcome of influenza (Figure 5). Specifically, among the relevant gut microbiota with anti-influenza benefits, down-regulation of Plasma Blast–Plasma Cell %lymphocyte mediated the protection of *Actinomycetota* against influenza, with a mediating effect ratio of 3.46%. Moreover, it also conferred protection against influenza and pneumonia through the same immune cells in which the mediating effect ratio was 2.88%. *Bifidobacterium* is part of *Actinomycetota*, with resistance to influenza, and it minimized the abundance of Plasma Blast–Plasma Cell %lymphocyte and Plasma Blast–Plasma Cell Absolute Count, which mediated 4.09% and 5.69% of the total effect, respectively. We identified a number of regulatory effects of peripheral immune cells that contributed to the higher mediating effects in the relationship between gut microbiota and influenza outcome. *Alcaligenaceae* and *Dorea* mediated their 21.10% and 18.55% protective effects against influenza by down-regulating the number of CD64 on CD14- CD16- cells, respectively. Additionally, *Dorea* exerted a positive effect on influenza by modulating the amount of HLA DR+ CD4+ T cell %T cells, which mediated 15.31% of the total effect. The phylum *Defluviitaleaceae* and the genus *Defluviitaleaceae* mediated 10.46% and 10.91% of their influenza protective effect by upregulating CD33+ HLA DR+ CD14- %CD33+ HLA DR+ cells. The effect of *Clostridia* against influenza and pneumonia was induced by up-regulating the IgD- CD24- B cell absolute count. Alternatively, for other gut microbiota with an elevated risk of influenza, immune cells were also engaged to that effect. The *Eubacterium ruminantium group* promoted influenza infection by increasing the number of CD25 on IgD+ CD24- B cells with a mediated effect of 4.07% (Figure 5).

## 3. Discussion

Host susceptibility to disease is generally heterogeneous, increasingly influenced by variations in gut microbiota. The vast array of microbiota in our gut is widely recognized for maintaining immune homeostasis and defending against pathogens [14]. Connections between the gut microbiota and distant organs form the basis of multiple regulatory mechanisms, such as the gut–lung axis, which mediates a wide range of chronic and infectious diseases [15,16]. Recent studies have highlighted the impact of gut microbiota–lung interactions on both acute and chronic respiratory diseases [17,18], and their role in the occurrence and progression of these conditions. In influenza, which causes varying degrees of respiratory disease, changes in gut microbiota can positively or negatively influence the infection [19,20].

Humans are complex organisms, and the interrelationship between gut microbiota and influenza virus infection is not a simple, unidirectional interaction, but a dynamic crosstalk [21]. Beneficial gut microbiota enhances host resistance to influenza by promoting innate antiviral immunity [22], suggesting that probiotics may hold the potential for prevention and therapy [23]. However, viral infections disrupt gut microbiota homeostasis, and the predominance of harmful bacteria exacerbates lung infections, potentially leading to sepsis in a vicious cycle [24]. Susceptibility to influenza can also increase when gut microbiota is damaged or depleted following antibiotic treatment [25].

An increasing number of researchers are focusing on the role of specific gut microbiota in influenza infections through systematic and focused investigations. Large-scale RCTs are costly and challenging, while cohort and retrospective studies often fail to exclude confounding factors. MR analysis, a genetic inference method that eliminates confounding effects, allows for the analysis of specific gut microbiota contributions to influenza risk [26]. Our study identified that gut microbiota implicated in influenza infections are mainly concentrated in the *Bacillota* and *Actinomycetota*, encompassing various genera. Among them, the *Bifidobacterium*, *Lachnospiraceae UCG010*, *Dorea* and *Defluviitaleaceae UCG011* were significantly associated with a lower risk of influenza onset. *Bifidobacterium*, a common commensal microorganism in the digestive tract, strengthens resistance to severe influenza infections [27]. This benefit may be derived from surfactant protein D (SP-D) in its cell wall, which is an innate sensor facilitating antiviral defenses [28]. SP-D neutralizes viral infectivity, inhibits viral neuraminidase activity, prevents viral entry into epithelial cells, and enhances phagocytosis and cleavage of influenza [29]. Both *Lachnospiraceae UCG010* and *Dorea* are involved in carbohydrate fermentation to produce short-chain fatty acids that maintain normal barrier function, correlating with the host’s healthy physiological state. A. Kaushal et al. observed that *Lachnospiraceae* levels in COVID-19 patients’ intestines were dramatically reduced and replaced by pathogenic microbes, leading to lung hyperinflammation [30]. *Defluviitaleaceae UCG011* contributes to metabolism and immunity, interacting with viral infections [31]. Conversely, the *Eubacterium ruminantium group*, *Romboutsia*, and *Anaerotruncus* were positively associated with influenza. Increases in these opportunistic pathogens indicate gut microbiota disturbance [32,33], with *Romboutsia* further promoting pulmonary fibrosis progression [34]. Notably, *Actinomycetota* and *Defluviitaleaceae UCG011* exhibited protective effects against influenza and pneumonia, suggesting their potential as probiotics for preventing secondary severe pneumonia post-influenza infection.

How does the gut microbiota impact influenza infection? There is growing clinical evidence that excessive inflammatory responses in the lungs (also known as “cytokine storms”) are strongly associated with high morbidity and mortality from influenza A viruses [35]. Targeted inflammatory therapy is an effective regimen for mitigating acute respiratory infections caused by influenza viruses and controlling the tissue damage resulting from excessive inflammation during acute viral infections. Influenza viruses enter the host through the upper respiratory tract and directly infect airway epithelial cells, alveolar epithelial cells, and immune cells. Viral infection triggers the cellular immune pathway, with neutrophils being the earliest mobilized cells in response and playing a crucial role in eliminating the virus. Monocytes deliver viral antigens to lymphocytes and activate T and B lymphocytes. Activated lymphocytes help eliminate the virus, produce antibodies, and express large amounts of inflammatory cytokines and chemokines in the respiratory tract, alveolar lumen and blood. Numerous studies have confirmed the crosstalk between gut microbiota and immune cells [36]. The commensal microbiota shape the function, development, and differentiation of immune cells to maintain host immune homeostasis. Dysbiosis of the gut microbiota leads to immunological imbalances; for instance, influenza A virus hemagglutinin triggers a widespread B-cell response [37], thus affecting the host’s capacity to handle influenza. In our study, we detected 65 immune cell types significantly associated with the risk of acquiring influenza or influenza and pneumonia (Figure 2). Mediation analyses revealed that multiple traits of immune cells played a crucial role in how gut microbiota regulated influenza infection. Notably, *Bifidobacterium* reduces pathogen stimulation by modulating the proliferation and differentiation of B cells [38]. This modulation is potentially mediated by the production of extracellular polysaccharides (EPS) [39], which promote symbiotic–host interactions, mediate immune evasion, and specifically modulate EPS-dependent B-cell responses. This enables *Bifidobacterium* to survive adverse conditions in the gastrointestinal tract and forms biofilms that protects the host from pathogen attack [40]. At the same time, a variety of soluble factors associated with gut microbiota induce the differentiation of activated B cells, with IFN producing non-antibody-secreting plasmablasts and IL-6 inducing their differentiation into antibody-secreting plasma cells [41]. Antibodies are produced by plasma cells, which are the final stage of B-cell differentiation [42]. It was found that ST6Gal I, a glycosyltransferase expressed by B cells, exhibited a high binding rate to plasmablasts and other cells after viral infection, and was involved in regulating the intrinsic antiviral immune response of B cells [43]. The balance of differentiation between plasmablasts and plasma cells will affect susceptibility to infection by the influenza virus. The *Eubacterium ruminantium group* influences specific B cell subsets via CD25 and IgD. CD25+ B cells, which exclude immunoglobulin secretion, show enhanced antigen presentation compared to CD25- B cells [44]. IgD B cells are age-associated B cells [45]. This has been shown to be a major pathway for the generation of memory B cells in aged mice infected with the influenza virus, inducing faster weight regain and producing higher total anti-influenza IgG and IgM titers that neutralize the virus [46]. HLA-DR, a major histocompatibility complex class II cell surface receptor, indicates monocyte activation and plays a critical role in initiating immune responses [47]. Both *Defluviitaleaceae UCG011* and *Dorea* interact with different monocyte subpopulations through HLA-DR, suggesting these microorganisms counteract influenza by modulating monocyte recruitment, activation, and subsequent T-cell immune responses. *Dorea* also regulates immune cells through CD64. CD64, the member of the FcγR family with the highest affinity for immunoglobulin G, is expressed on macrophages, neutrophils, and eosinophils [48]. Macrophages are the main source of type I IFN in viral infections, forming FcγRI–antibody–pathogen complexes during pathogen infection and mediating a series of downstream inflammatory immune responses. CD64 is mobilized by microbial cell wall components, complement division products, and pro-inflammatory cytokines such as interferon γ (IFN-γ) and granulocyte colony-stimulating factor (G-CSF). Its expression may differentiate between infected and non-infected individuals at early stages [49]. These findings underscore the significant relationship among gut microbiota, immunity, and lung infections.

## 4. Method

### 4.1. Study Design

**Figure 6:**
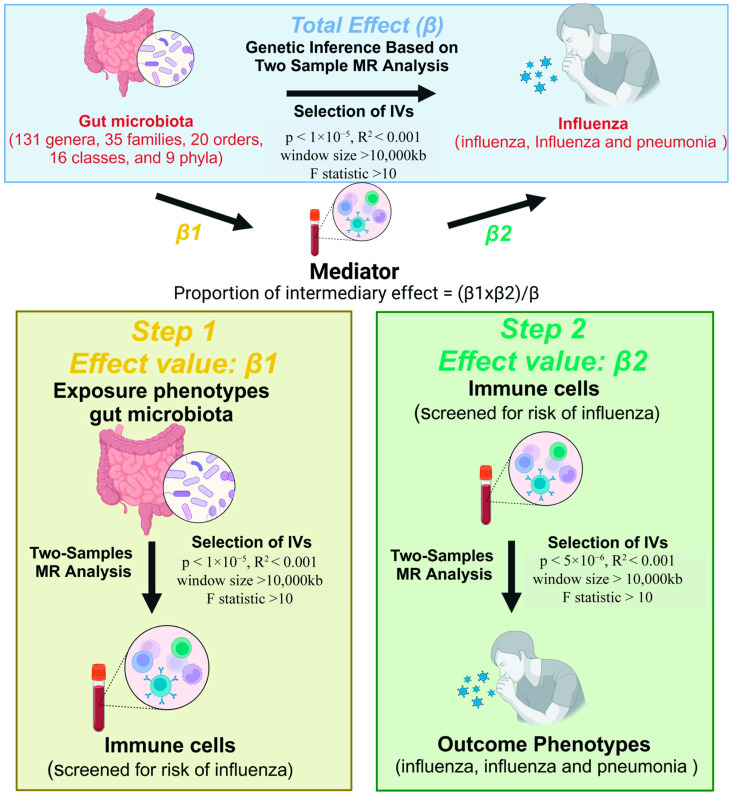
Analysis process (created with Biorender).

First, we analyzed the causal association of 211 gut microbiota with influenza outcomes (influenza and influenza combined with pneumonia) using two-sample MR in such a way that specific gut microbiota associated with influenza outcomes were screened. Next, to explore the mediating role of peripheral immune cells, peripheral immune cell species associated with influenza outcomes were first analyzed by two-sample MR analysis. Further, two-sample MR analysis was used to determine the effect of gut microbiota on influenza-associated peripheral immune cell species. Finally, a two-step approach was used to determine the mediating role of peripheral immune cells in the causal inference of gut microbiota and influenza susceptibility (Figure 6).

### 4.2. Exposure and Outcome Phenotypic Data

The gut microbiota phenotypic data were derived from the largest current sample size of the GWAS study of gut microbiota in humans conducted by the MiBioGen consortium. This study enrolled a total of 24 cohorts with 18,340 participants, primarily from Europe [50]. GWAS studies related to peripheral immune cells were derived from a clinical cohort study that included 3757 Sardinians, collectively identifying whole genome profiles containing 731 species of peripheral immune cells [51]. The FinnGen research program, which began with a large whole genome association analysis in 2017, enrolled more than 500,000 Finns with genomic information and related biological data [52]. Influenza-related GWAS data were obtained from the FinnGen consortium for phenotypes including “influenza” and “influenza and pneumonia”. These data are available directly through the IEU Open GWAS program.

### 4.3. Instrumental Variables Selection

Rigorous screening conditions for instrumental variables served to guarantee the reliability of analysis results. Following the criteria of a previous study [53], for the raw GWAS data, *p* < 1 × 10^−5^ was used to select single nucleotide polymorphism (SNPs) associated with 211 species of gut microbiota, while *p* < 5 × 10^−6^ was used for 731 species of peripheral immune cells, which will be used as a tool for genetic inference in the next analysis. Since linkage disequilibrium among IVs biases the analysis results, R^2^ < 0.001 and a window size >10,000 kb were used to eliminate SNPs with linkage disequilibrium. Furthermore, the presence of weak IVs also interfered with the results, and according to the calculation method in the previous study [54], we performed an F-statistic calculation on the screened SNPs. If the value is <10, the SNPs will be excluded.

### 4.4. MR Estimate

Two-sample MR was applied to analyze the causal relationship between exposure and outcome phenotype, involving inverse-variance weighted (IVW), MR Egger, and Weighted median. Of these, IVW is the main method, which assumes that it is performed with all instrumental variables being valid, and *p*_IVW_ < 0.05 was used to determine the existence of a causal relationship. While MR Egger and Weighted median were considered as complementary methods, they assumed that the analysis was performed with all or half of the IVs as invalid [52]. Estimated effect sizes were expressed by odds ratio (OR, 95% CI) for the categorical data (onset of influenza), and β (95% CI) for the measurement data (immune cell count).

### 4.5. Sensitivity Analysis

The presence of horizontal pleiotropy would seriously undermine the reliability of MR analysis, so we made no causal inferences about the results with horizontal pleiotropy. For horizontal pleiotropy analysis, the Egger intercept test was used to capture the presence or absence of pleiotropy. Also, Cochran’s Q test was used to verify the presence of heterogeneity in the analysis results. The leave-one-out analysis was finally used to test the robustness of the results.

### 4.6. Mediation Effect Analysis

According to reference [55], immune cell phenotypes included for mediator analyses are expected to satisfy the following conditions: (i) the exposure factor has only a unidirectional effect on the mediator; (ii) the direction of the mediator–mediated indirect effect is supposed to be the same as the direction of total effectivity from exposure to the endpoint; (iii) there were significant effects between exposure on mediator and mediator on outcome, with the effect of exposure on mediator being unidirectional. The coefficient product method was used to calculate the proportion of mediator effect. All the above analyses were conducted by R software (4.4.0) and the Two Sample MR package (0.6.1). The process of analysis is shown in Figure 6.

#### 4.6.1. Filtering for Influenza-Associated Gut Microbiota

Two-sample MR analysis was used to assess the total effect of gut microbiota to influenza-associated phenotypes. For results with significant associations, the effects of influenza on these gut microbiota were assessed by inverse MR analysis. The positive effects of these were included in the next step of mediated effects analysis.

#### 4.6.2. Identification of Influenza-Associated Immune Cells

We assessed the effects of 731 species of peripheral immune cells on influenza phenotypes, and enrolled immune cells with significant effects into the next step of mediated effects analysis.

#### 4.6.3. MR Analysis of the Two-Step Method

In the mediation analysis of the two-step approach, the total effect of gut microbiota on the influenza outcome was β, the effect of gut microbiota on the mediator was β1, the effect of the mediator on the influenza outcome was β2, and the proportion of the mediating effect was β1 * β2/β (Figure 6).

## 5. Conclusions

Overall, the purpose of this work was to characterize the composition of the microbiota relevant to influenza-associated respiratory infections and systemic immunity. These results not only enriched our understanding of the anti-influenza impact of gut microbiota but also provided new insights into host-microbiota interactions. Gut microbial species or oral probiotics interact with immune cells in various ways, both directly and indirectly. Understanding these interactions is crucial for developing the theoretical bases for the beneficial effects of orally relevant probiotics and for designing gut microbiota and immune cell intervention strategies targeting influenza therapies. However, our study has limitations. It was based on a GWAS study from European populations only, and results from other populations need cautious analysis. Additionally, we could not stratify the study by age due to limitations in the original GWAS data, even though influenza is particularly severe in older adults and children.

## Data Availability

GWAS data on influenza, gut microbiota taxa, and peripheral immune cells are publicly available and accessed through the IEU open GWAS project database (https://gwas.mrcieu.ac.uk/) (accessed on 5 May 2024).

## Supplementary Materials

The following supporting information can be downloaded at: https://www.mdpi.com/article/10.3390/ijms25147706/s1.
